# Lipid Shape as a Membrane Activity Modulator of a Fusogenic Antimicrobial Peptide

**DOI:** 10.1021/acs.jcim.4c02020

**Published:** 2025-03-20

**Authors:** Marcin Makowski, Octávio L. Franco, Nuno C. Santos, Manuel N. Melo

**Affiliations:** 1 GIMM − Gulbenkian Institute for Molecular Medicine, Av. Prof. Egas Moniz, Lisbon 1649-035, Portugal; 2 Faculdade de Medicina, Universidade de Lisboa, Av. Prof. Egas Moniz, Lisbon 1649-028, Portugal; 3 Instituto de Tecnologia Química e Biológica António Xavier, 50106 Universidade Nova de Lisboa, Oeiras 2780-157, Portugal; 4 Facultad de Ciencias Químicas, Departamento de Química Física, Universidad Complutense de Madrid, Avda. Complutense s/n, Madrid 28040, Spain; 5 Instituto de Investigación Biomédica Hospital Doce de Octubre (imas12), Avenida de Córdoba s/n, Madrid 28041, Spain; 6 Instituto Pluridisciplinar, Paseo Juan XXIII 1, Madrid 28040, Spain; 7 Programa de Pós-Graduação em Patologia Molecular, Faculdade de Medicina, Universidade de Brasília, Campus Darcy Ribeiro, Asa Norte, Brasília, Distrito Federal 70910900, Brazil; 8 Centro de Análises Proteômicas e Bioquímicas, Pós-Graduação em Ciências Genômicas e Biotecnologia, Universidade Católica de Brasília, SGAN 916 Módulo B, Asa Norte, Brasília, Distrito Federal 70790160, Brazil; 9 S-inova Biotech, Programa de Pós-Graduação em Biotecnologia, Universidade Católica, Dom Bosco Avenida Tamandaré 6000, Campo Grande, Mato Grosso do Sul 79117900, Brazil

## Abstract

An intriguing feature of many bacterial membranes is their prevalence of non-bilayer-forming lipids, such as the cone-shaped phosphatidylethanolamines and cardiolipins. Many membrane-active antimicrobial peptides lower the bilayer-to-hexagonal phase transition energy barrier in membranes containing such types of cone-shaped lipids. Here, we systematically studied how the molecular shape of lipids affects the activity of antimicrobial peptide EcDBS1R4, which is known to be an efficient fusogenic peptide. Using coarse-grained molecular dynamics simulations, we show the ability of EcDBS1R4 to form “hourglass-shaped” pores, which is inhibited by cone-shaped lipids. The abundance of cone-shaped lipids further correlates with the propensity of this peptide to oligomerize preferentially in antiparallel dimers. We also observe that EcDBS1R4 promotes the segregation of the anionic lipids. When coupled to dimerization, this charge segregation leads to regions in the bilayer that are devoid of peptides and rich in zwitterionic lipids. Our results indicate a protective role of cone-shaped lipids in bacterial membranes against pore-mediated permeabilization by EcDBS1R4.

## Graphical abstract

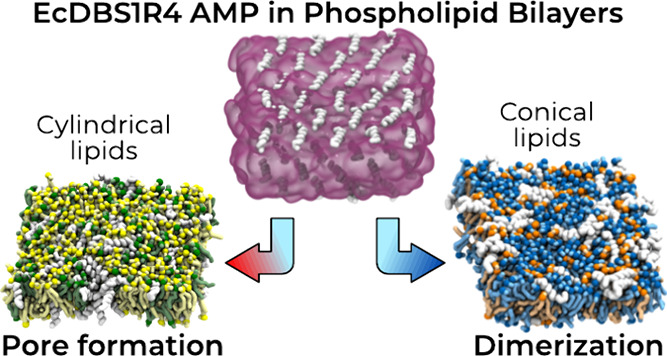

## Introduction

The discovery rate of new antibiotic molecules plummeted three decades ago, and bacterial pathogens are becoming resistant to every known antibiotic class.^1^ Alternatives to conventional antibiotics are required to prevent the uncontrolled spread of antimicrobial resistance. Among the most promising drug weapons that could help control antimicrobial-resistant infections are antimicrobial peptides (AMPs).^2,3^ These amphipathic immune effector molecules kill targeted organisms by different mechanisms, but chiefly by destabilizing the structural integrity of their cell membranes.^4^ The molecular processes by which AMP activity causes membrane collapse have been the subject of extensive research.^5–7^ Various general models have been proposed, including forming pores, detergent-like mechanisms, sequestration of anionic lipids, and mechanisms involving intracellular targets.^8^

The physical-chemical properties of both the peptide and the lipid bilayer^9,10^ frequently determine the mechanism of action of an AMP. While a net-positive charge favors the interaction of the AMPs with the membranes of bacteria—which are enriched in anionic phospholipids—peptide hydrophobicity allows insertion and dictates the collective behavior of AMPs, such as pore formation.^11^ The physical-chemical properties of the phospholipid bilayer, in turn, modulate the mode of action of AMPs.^10,12,13^ For instance, the membrane-disruptive activity of AMPs often involves the generation of curvature. The propensity of the lipid bilayer to adopt such curvatures is influenced by the molecular shape of its lipid constituents, and such tendency can be quantified through the lipid intrinsic curvature (*c*_0_).^14^ Cylinder-shaped lipids have similar cross-sectional areas at the headgroup and the aliphatic chain level, readily forming bilayers when mixed in an aqueous suspension. These cylinder-shaped lipids are said to have zero intrinsic curvature (*c*_0_ = 0). In contrast, lipids with tails bulkier than headgroups, known as cone-shaped, are not prone to adopting lamellar phases in solution and are said to have negative intrinsic curvature (*c*_0_ < 0).^15^ Curiously, the plasma membrane of Gram-negative bacteria such as *Escherichia coli* has more non-bilayer-forming negative intrinsic curvature phospholipids (phosphatidylethanolamine and cardiolipin) than bilayer-stabilizing ones.^16^ This enrichment in nonbilayer-forming lipids may be due to their role in the assembly, stabilization, and function of membrane proteins.^17,18^ Essential biological functions involving the deformation of membranes—such as cell division, budding, or membrane fusion—also require non-bilayer-forming lipids.^19^

In this work, we employed the “computational microscope”^20^ of coarse-grained molecular dynamics simulations (CG-MD) to explore how lipid shape can modulate the activity of a model AMP. CG-MD can provide unique perspectives into the mechanism of membrane-active peptides.^21^ The peptide of choice was EcDBS1R4 (PMKKKLAARILAKIVAPVW), a designed AMP effective against *E. coli* from a family of rationally designed bioinspired peptides, recently explored as templates for new antimicrobials.^22–24^ EcDBS1R4 bears several representative characteristics of the AMP class: it has a net-positive charge, abundant hydrophobic residues, and transitions from a disordered to an α-helical secondary structure in the presence of anionic lipids.^25^ However, previous reports suggest that the *in vivo* activity of EcDBS1R4 does not involve the formation of long-lasting pores, as it does not depolarize the membrane of *E. coli*.^25,26^ Additionally, EcDBS1R4 promotes the hemifusion of biomembrane models mimicking the lipid composition of the inner membrane of *E. coli*, suggesting that it favors a bilayer-to-hexagonal phase transition, as required for the hemifusion stalk. We simulated several membranes, varying the proportion of cone-shaped lipids, and were able to shed light on the cryptic behavior displayed by this peptide *in vitro*. Namely, we identified a dependency of EcDBS1R4 membrane activity on the environment: lipid bilayers lacking cone-shaped lipids promote long-lasting transmembrane contacts between peptides from opposite sides of the bilayer that evolve into pore structures; in contrast, more significant concentrations of cone-shaped lipids induce long-lasting peptide dimers but inhibit transmembrane contacts between peptides and the formation of pore structures.

## Results

### Cone-Shaped Lipids Inhibit EcDBS1R4 Pore Formation

To investigate the effect of lipid shape on the membrane activity of EcDBS1R4, we prepared membrane systems with diverse proportions of cylinder- (POPC, 1-palmitoyl-2-oleoyl-*sn*-glycero-3-phosphocholine, and POPG, 1-palmitoyl-2-oleoyl-*sn*-glycero-3-[phospho-*rac*-(1-glycerol)]) and cone-shaped lipids (POPE, 1-palmitoyl-2-oleoyl-*sn*-glycero-3-phosphoethanolamine, and CL, tetraoleoyl-cardiolipin), while keeping a constant net charge density in the membrane (Figure 1 and first four systems in Table 1). Maintaining constant the proportion of anionic lipids in the bilayer—in which we counted CL, with its four tails and two headgroup phosphates, as equivalent to two anionic lipids—was crucial to avoid introducing charge density as an additional variable influencing the system. Lipids with negative charges are found in the bacterial membranes. As they are frequently the target of AMPs, these lipids may modulate their selectivity and activity. To ensure that membrane activity was not limited by the peptide concentration in the lipid bilayer, a constant and high peptide-to-lipid ratio (P/L ratio) of 1:12 was maintained in all lipid bilayers, with peptides added to both leaflets. This ratio is a reasonable estimate considering a partition coefficient (*K*_p_) of 20 × 10^4^, the experimentally determined minimum inhibitory concentration (MIC) of 11 μM,^25^ and an average lipid molar volume (γ_*L*_) of 0.8 L mol^–1^, which together can be related to the L/P ratio through the relation:^27^

$$P/L={\mathrm{MICK}}_{p}\gamma_{L}$$

**Table 1:** Summary of Simulated Membrane Systems

lipid system	proportion	P/L ratio	replicates	total simulation time (μs)
POPC/POPG	2:1	no peptide	3	60
		1:12^a^	3	60
POPC/CL	4:1	no peptide	3	60
		1:12	3	60
POPE/POPG	2:1	no peptide	3	60
		1:12	3	60
POPE/CL	4:1	no peptide	3	60
		1:12	3	60
POPE/POPG/CL	65:30:5	no peptide	3	60
		1:12	3	60
POPG/CL^b^	4:1	no peptide	3	60
		1:12	3	60
POPC^b^		no peptide	3	30
		1:12	3	30
POPE^b^		no peptide	3	30
		1:12	3	30

a Systems in which hourglass-shaped pores were observed.

b Systems corresponding to additional controls to test specific hypotheses.

We observed spontaneous transmembrane contacts (i.e., contacts between peptides that were originally placed in opposing monolayers) of EcDBS1R4 in POPC/POPG (2:1) lipid bilayers. These contacts evolved into hourglass-shaped pore structures involving several peptides, with a central part wide enough to allow some solvent flow (Figures 1 and 2A and Supplementary Movie 1). The core of this hourglass-shaped pore is composed of the C-terminal residues of the peptides establishing interleaflet contacts, as mapped in Figure 2B. In this arrangement, the hydrophobic residues of the peptides participating in the hourglass-shaped pore face the lipid acyl tails, leaving the positively charged residues exposed to the solvent (Figure 2A). Transmembrane contacts were observed in all three replicates of the POPC/POPG (2:1) composition. However, the duration of these contacts varied widely: while in two replicates transmembrane contacts were long-lasting (>1 μs) and resulted in pore formation, in the other replicate, they were short-lived, occurred only twice, and did not lead to the formation of a pore structure. In further analyses, we used the latter as a no-pore control system. Replacing the cylinder-shaped POPG with the cone-shaped CL resulted in no pores or transmembrane contacts. Similarly, replacing the cylinder-shaped zwitterionic POPC with the zwitterionic but cone-shaped POPE also inhibited pore formation. Anionic lipids were not necessary to form hourglass-shaped pores, as these spontaneously assembled when in pure POPC bilayers, which lacked the anionic component. No pores or transmembrane contacts were observed in pure POPE bilayers under identical conditions.

### Dependence of EcDBS1R4 Dimerization on Lipid Shape

Inspection of the simulation trajectories showed that in lipid bilayers with compositions resembling that of *E. coli*’s inner membrane (with an enrichment in the cone-shaped POPE), EcDBS1R4 frequently and spontaneously self-assembled into membrane-adsorbed dimers. By contrast, dimers were less frequent in membranes where the zwitterionic but cylinder-shaped POPC replaced the zwitterionic cone-shaped POPE. The time-averaged interpeptide contact map with a distance threshold of 6 Å (Figure 2A) indicates that dimers had an antiparallel configuration, with the residues Lys4 of one peptide and the terminal tryptophan (Trp19) of the other being the most frequent contacts. Therefore, this pair of residues was chosen as a dimer reporter to study the lifetimes of dimerization (Figure 2B): a peptide pair was considered a dimer only when both peptides were contacting each other via the Lys4–Trp19 residue pair. The dimerization lifetime behavior in most compositions can be described as bimodal, with a subpopulation of short-lived dimers (lasting less than 50 ns) and a long-lived subpopulation (lasting more than 400 ns).

**Figure 1:**
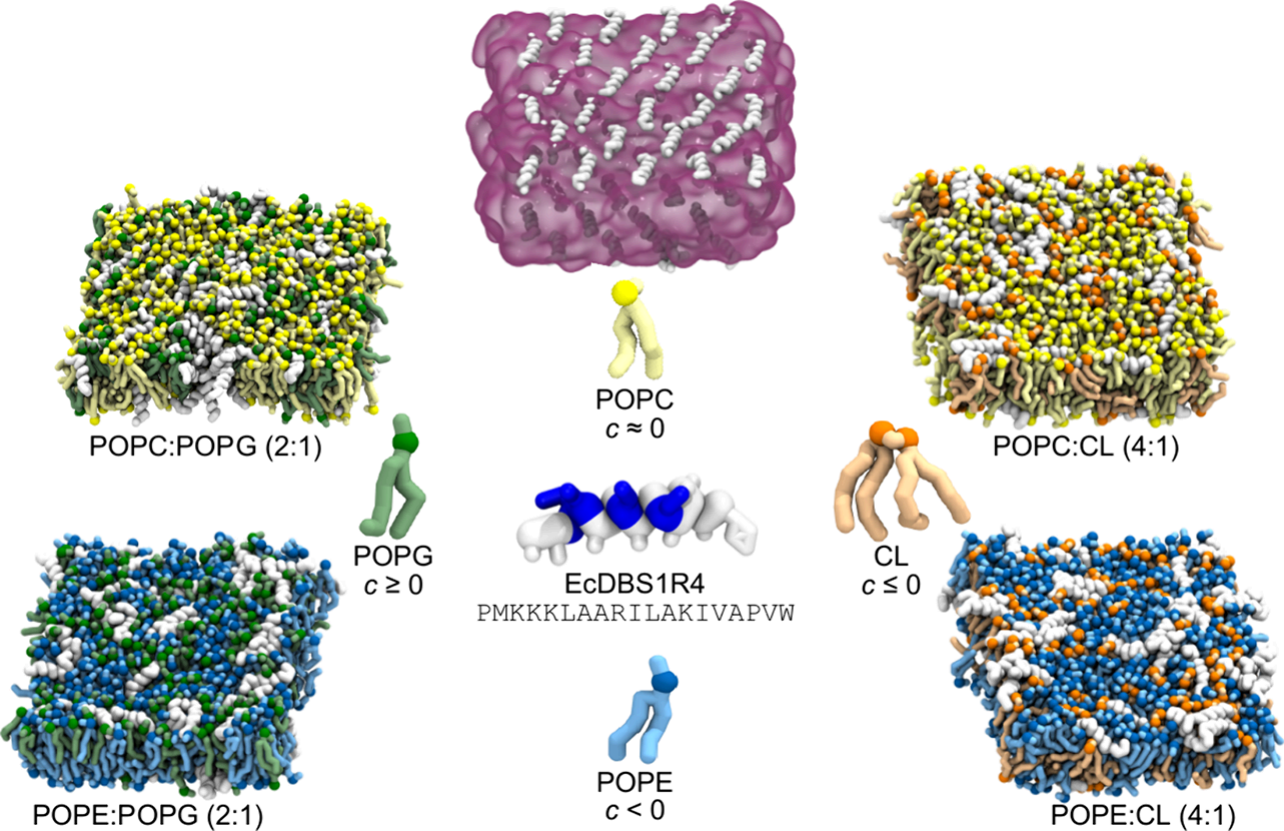
Coarse-grained-representation of the main simulated systems. Top: representative snapshot of the configuration after the bilayer adsorption process, with the lipid bilayer represented in translucent magenta and the peptides—which start out in parallel orientations—in white (note the checkered pattern in the initial disposition of peptides). Left and right are snapshots with the final configuration of the main simulated systems. Lipids are color-coded according to the representation displayed in the center, while peptides are in white. Phosphate beads are represented as spheres. This color code is maintained in the remaining figures. For each lipid, the sign of its intrinsic curvature, *c*, is indicated below its name. The central coarse-grained representation of EcDBS1R4 peptide has the residues colored according to polarity/charge: white, apolar; basic, blue. The compositions and simulation times of all simulated membrane systems are detailed in Table 1.

**Figure 2:**
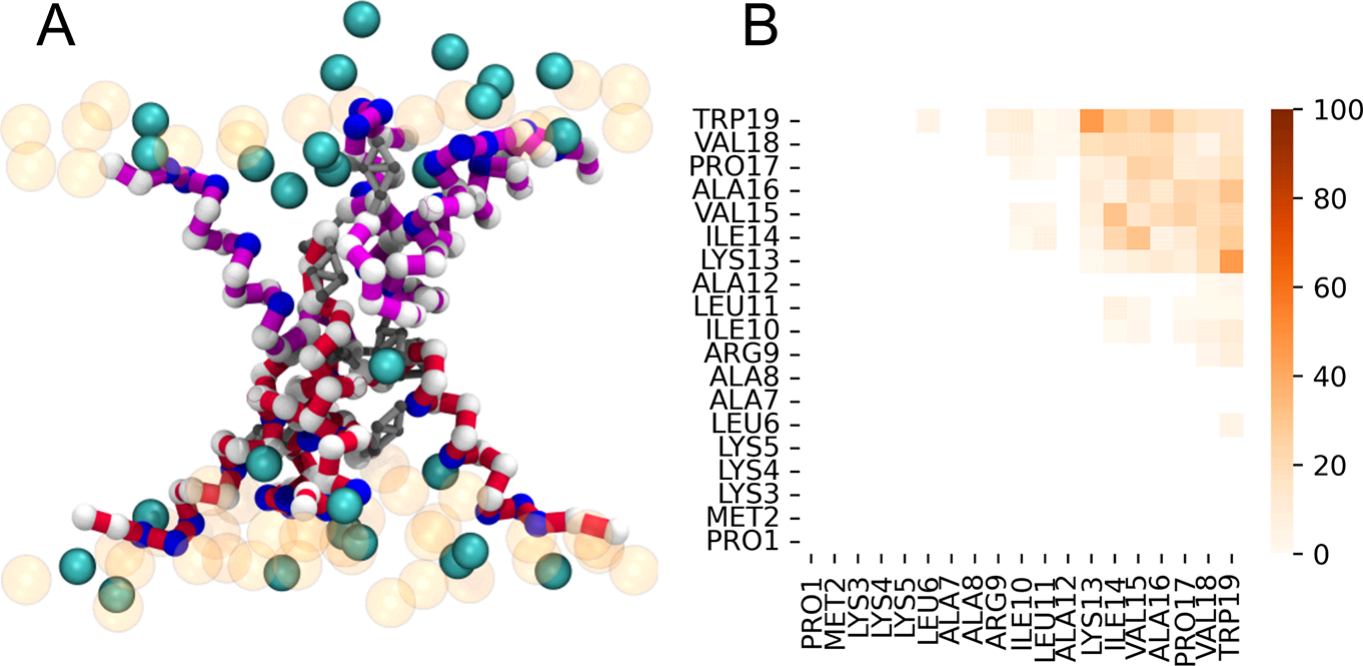
EcDBS1R4 hourglass-shaped pore structures in membranes lacking cone-shaped lipids. (A) Snapshot depicting the hourglass-shaped structure observed in a POPC/POPG (2:1) bilayer. Backbone bonds of intervening peptides are in magenta and crimson (for peptides initially placed in the top and bottom bilayer, respectively). Backbone beads are white (hydrophobic residues) and blue (basic residues). Only the phosphate group of each lipid is shown in translucent orange. Waters in the pore vicinity are shown in cyan. (B) Time-averaged transmembrane peptide contact map indicating the preferred residue–residue contacts established during pore formation. The color scale indicates the relative contact prevalence as a percentage over the analyzed pore frames.

In Figure 3A, the different lipid bilayers are shown in ascending order of total dimerization time, which coincides with the ascending proportion of cone-shaped lipids in that bilayer. Mixtures containing POPE show the longest-lasting dimers, with survival times frequently exceeding hundreds of nanoseconds up to the microsecond range. In contrast, lipid mixtures containing POPC had significantly shorter-lived dimers; however, replacing POPG with CL in these mixtures significantly increased dimer survival time. Again, this indicates that cone-shaped lipids influence the propensity toward dimerization.

**Figure 3:**
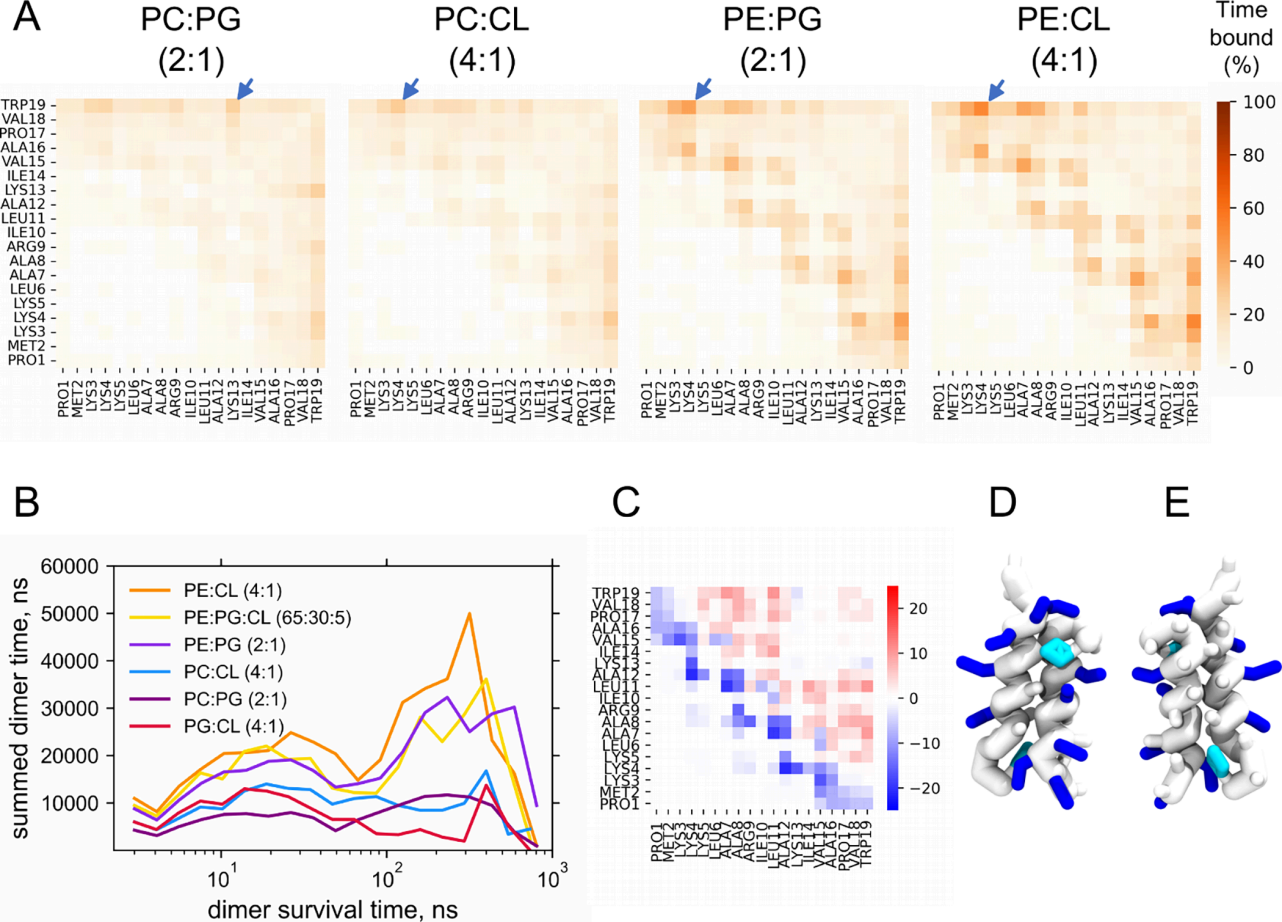
EcDBS1R4 dimerization tendency in different lipid compositions. (A) Time-averaged peptide–peptide contact maps indicating the frequency of peptide residue contacts within the same leaflet. Arrows point at the most frequently contacting pair of residues. (B) Dimer lifetime histogram depicting the summed dimer time (sum of the time that peptides spent as dimers) as a function of the dimer survival time. (C) Comparative view between the peptide–peptide contact maps in POPE/CL (4:1) and in pure POPE bilayers. Residues colored red indicate contacts that were more frequent in the POPE/CL (4:1) composition. Blue-colored residues indicate the converse. (D,E) Snapshots depicting the view from the solvent (D) or from the center of the bilayer (E) of an EcDBS1R4 dimer in a POPE/CL (4:1) bilayer, showing the side chains of basic residues in blue and of hydrophobic residues in white. As a guide, Trp19 side chains are colored in cyan.

We probed whether lipid charge also influences peptide dimerization by monitoring EcDBS1R4 behavior in bilayers composed of only anionic lipids (POPG/CL (4:1)) and pure zwitterionic POPE bilayers. In the latter, dimers were abundant and long-lasting (Supplementary Figure 1). In contrast, dimers in the POPG/CL (4:1) bilayer had the lowest survival times. This suggests that (i) the lipid cone shape is key for the establishment and durability of dimers and (ii) the presence of zwitterionic lipids with such shape is needed for a long-lasting peptide dimerization. Interestingly, the dimer contact map in pure POPE has a distinct pattern from that observed in bilayers with anionic lipids (Figure 3C), with dimers in pure POPE closer to antiparallel conformations in complete alignment (overall maintaining the same interface but shifted by one turn relative to the dimers in the other systems).

We analyzed the dependence of peptide self-assembly on its insertion angle in the different bilayers (Supplementary Figure 2). Tilt angle distributions of EcDBS1R4 displayed a mean value close to zero, but in all compositions slightly negative (i.e., the C-terminus shallower than the N-terminus), possibly due to the anchoring effect of the tryptophan residue^28,29^ or the segregation of cationic lysine/arginine residues along an irregular patch. Peptide dimerization had little effect on the angle of insertion of EcDBS1R4. Conversely, self-assembly of peptides into hourglass-shaped pores involved a positive tilt angle of the peptide (i.e., with the C-terminus region buried; see also Figure 2A).

### Bilayer Thinning Induced by EcDBS1R4

We investigated how the different lipid bilayers influenced the peptide’s insertion depth and how the lipid bilayer’s thickness responded to the inserted peptides. We calculated the average Z-distances of the lipid phosphates relative to the center of the bilayer. In the absence of peptide, the system with the thinnest lipid leaflets was POPC/POPG (2:1), followed by POPE/POPG (2:1): 19.9 and 20.3 Å, respectively (Figure 3A). Conversely, bilayers containing CL were thicker, yielding 21.5 Å for POPE/CL (4:1) and 21.1 Å POPC/CL (4:1). The increase in thickness imposed by CL may be explained by the particular Martini 2 model that was used: Martini 2 CL has an extra particle (or “bead”) in its oleoyl chain (5 beads vs the 4 beads of the palmitoyl-oleoyl phospholipids POPC, POPE, or POPG). Lipid species accompanying CL generally protruded higher, suggesting that the longer tails of CL lead to the stretching of shorter lipids in their vicinity for better hydrophobic thickness matching.

Interaction of the peptide with the bilayer caused bilayer thinning in all cases, with the extent of thinning being dependent on the lipid composition (Figure 4). To better understand how peptide incorporation affected lipid bilayer thickness, we plotted the ratio of phosphate Z-distances between the simulations with and without peptide (Z_PEP+_/Z_PEP_–; Figure 4B). The most responsive lipid was POPE, which became almost 10% thinner, followed by POPC, with close to 6% thinning. Anionic phospholipid thickness was less sensitive: in general, POPG and CL were less affected than their zwitterionic partners, except for the POPC/POPG (2:1) composition, where both lipids had similar thicknesses and were affected similarly by EcDBS1R4.

**Figure 4:**
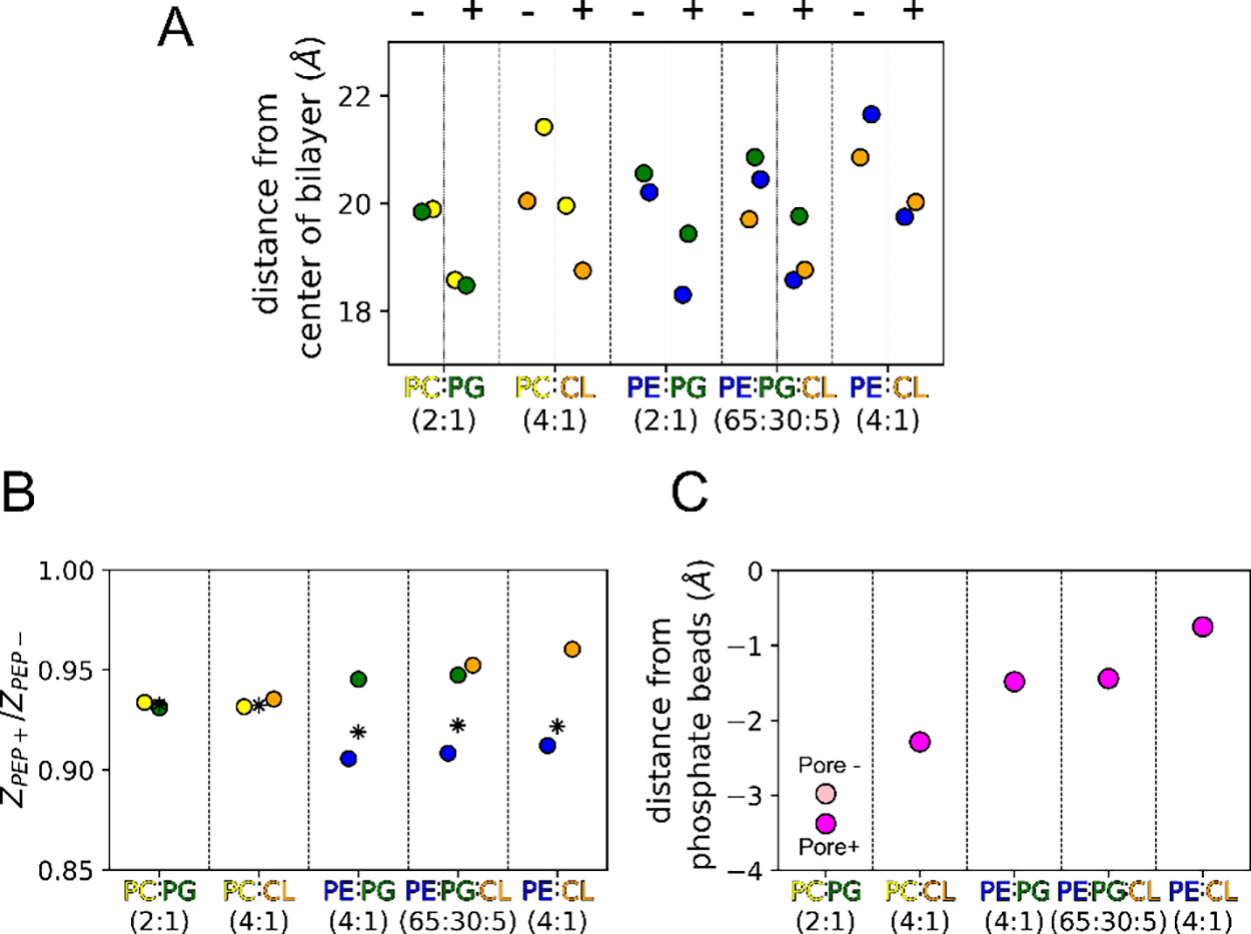
Effect of EcDBS1R4 on bilayer thickness and preferred peptide positioning along the bilayer normal in different membrane environments. (A) Average position of the phosphate beads along the bilayer normal in the absence (−) and presence (+) of peptide. (B) Ratio of position of the phosphate beads along the Z-axis in the presence and absence of peptide (PEP+/PEP−). (C) Distance of the peptide’s center of mass from the phosphate beads’ average position in each bilayer. Asterisks represent the means of the Z-distances of the phosphate beads for all lipids in the bilayer. Error bars (representing the CI 95%) are smaller than the symbol size (see Supplementary Figure 3). Lipid color codes are orange for CL, yellow for POPC, blue for POPE, and green for POPG.

The in-depth positioning of the backbone beads of the peptide along the bilayer normal depended on the composition. We measured the Z-distance between the peptides’ backbone particles and the average Z-position of the surrounding lipids’ phosphate groups (at a maximum distance of 10 Å) from the peptide (Figure 4C). In the case of POPC/POPG (2:1), the average peptide backbone position was the furthest from the phosphate region (3.4 Å deeper than that of the phosphate beads). This was true even when the depth was calculated by excluding the deeper hourglass-shaped transmembrane structures that were formed (3.0 Å). POPC/CL (2:1) had the next deeper peptide-diving (2.3 Å). By contrast, POPE/CL (4:1) was the composition in which the peptide positioned closest to the phosphate groups (0.7 Å deeper), followed by POPE/POPG/CL (65:30:5) and POPE/POPG (2:1), which were 1.4 and 1.5 Å deeper, respectively.

### EcDBS1R4 Effects on the Lateral and Transversal Order of the Bilayer

We also measured the area per lipid (ApL) for each lipid species. ApL reflects lipid packing, a parameter that can influence the membrane activity of AMPs.^30^ We employed a two-dimensional Voronoi tessellation-based method^31^ to obtain the ApL per lipid species. To have a comparable view on the global lipid packing of the bilayers, we measured the ApL of each lipid species and then performed the weighted average per pair of acyl chains to account for the difference in the number of acyl chains between CL (4) and the other lipids (2) (Figure 5A and Supplementary Figure 4A).

In the absence of peptide, the highest average ApL—and thus the least packed—was that of POPC/POPG (2:1). Increasing the proportion of cone-shaped lipids resulted in increased packing, with POPE/CL (4:1) being the most packed composition (with the lowest average ApL). To evaluate the impact of peptide addition on lipid packing, we obtained the ratio between the ApLs in the presence and absence of peptide (ApL_PEP+_/ApL_PEP_−) in the whole bilayer (Figure 5B), as well as at two distinct membrane environments: (i) the bulk (i.e., lipids not in close interaction with the peptide; Figure 5C) and (ii) the annular (i.e., lipids in contact with the peptide, within a cutoff distance of 1 nm; Figure 5D). Annular lipids experienced an increase in the ApL, especially the anionic ones (Figure 5B), with little variation between the tested compositions. On the other hand, bulk lipids experienced an increase in their packing in the presence of peptides, and this increase correlated positively with the membrane content of lipids with negative intrinsic curvature (Figure 5D). We also compared the ApL of annular lipids of dimers vs monomers (Supplementary Figure 4B). Dimers expanded further the ApL of all lipids, but especially so in the case of anionic species. The pore structure had an even stronger effect on the occupancy of surrounding lipids. This could reflect a bending of the tails to adapt to the tilted, transmembrane, peptide configurations, but could also reflect a less comparable area measurement due to the use of pore peptide particles to define the Voronoi cells.

In addition to in-plane bilayer packing, we monitored how the order along the bilayer normal responded to the presence of peptide presence. We calculated the second rank-order parameter, P_2_, for each of the bonds of the acyl chains, with P_2_ = 1 representing perfect alignment with the bilayer normal, P_2_ = −0.5 perfect antialignment, and P_2_ = 0 reflecting a random orientation. P_2_ was measured in the absence and presence of the peptide. In the absence of peptide (Supplementary Figure 5A), the POPC/POPG (2:1) mixture was the lipid composition with the lowest average acyl chain bond order (C1–C4), followed by POPC/CL (2:1), POPE/POPG (2:1), and POPE/CL (4:1). The presence of the peptide-induced change in acyl chain bond order was observed for all lipid compositions (Supplementary Figure 5B), with the extent of ordering/disordering depending on lipid composition, lipid type, position of the bond, and peptide-lipid distance. Noticeably, for POPC/POPG (2:1), EcDBS1R4 caused a significant increase in the orientation order of bonds located closer to the bilayer interface. Conversely, in all compositions, bonds closer to the bilayer core experienced a loss of order in the presence of EcDBS1R4. As the proportion of cone-shaped lipids increased, the bond orientation order of the acyl chain beads concomitantly decreased at all depths in response to EcDBS1R4. For most lipid compositions, the difference in acyl chain bond order between annular and bulk lipids (more disordered for the annular lipids) was pronounced for the more profound beads but negligible for bonds closer to the interface. Exceptionally, for POPE, the difference between annular and bulk bond orientation order was apparent from the first bond onward. This observation and the ApL data suggest that this cone-shaped lipid could contribute to a buffering effect over the packing stress caused by the AMP.

**Figure 5:**
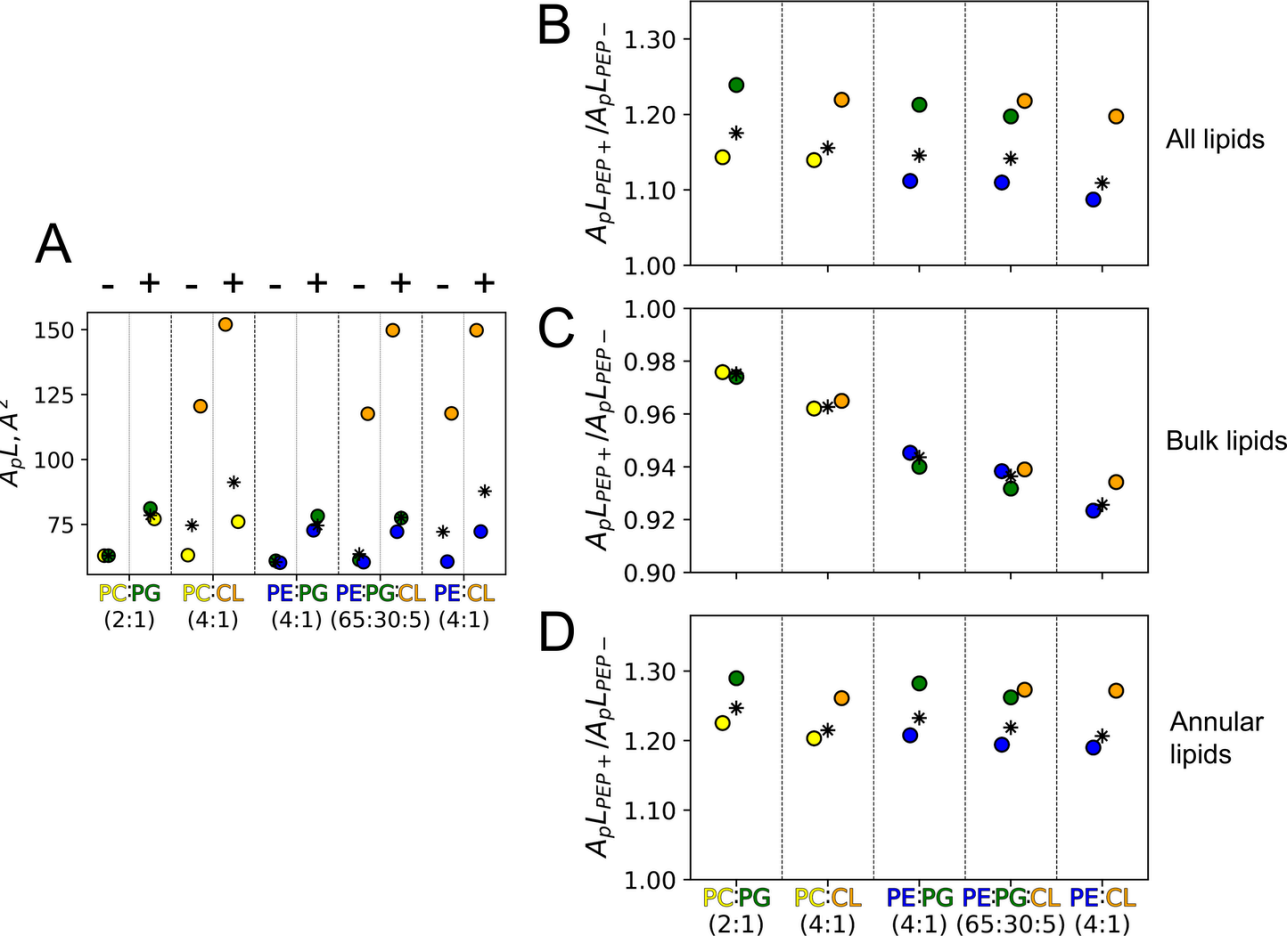
Effects of EcDBS1R4 on lipid packing. (A) ApL of all lipids for each lipid composition in the absence (−) and presence (+) of EcDBS1R4. Error bars (representing the CI 95%) are smaller than the symbol size (each replicate’s averages are plotted in Supplementary Figure 4A) (B–D) Ratios between the ApL (of all lipids) in the presence and absence of EcDBS1R4 in different lipid environments: all lipids in the bilayer (B), bulk lipids that are not in contact with peptide molecules (C), and annular lipids interacting with the peptide (D). Lipid color codes are orange for CL, yellow for POPC, blue for POPE, and green for POPG. Asterisks represent the weighted mean of the ApL ratios for all lipids in the bilayer.

### EcDBS1R4 Impact on Lateral Diffusion of Lipids of Different Shape

Lipids in biomembranes frequently self-organize laterally in domains of distinct dynamic properties—a key feature that has been posited to modulate the function of membrane proteins.^32^ We evaluated the effect of peptide insertion on lipid dynamics by measuring the lipid and peptide lateral diffusion coefficients (*D*) in the simulated systems. In the absence of peptide, CL was the lipid with the slowest lateral diffusion (Figure 6A), while POPG was the fastest diffusing lipid. On average, the slowest diffusing bilayer was POPC/CL (4:1), followed closely by POPE/CL (4:1).

The addition of the peptide slowed lipid lateral diffusion in all bilayers, affecting lipid species in different environments unevenly. To better understand the impact of EcDBS1R4 on the diffusion of each lipid species, we calculated the ratios of the diffusion coefficients with and without peptide (*D*_PEP+_/*D*_PEP_−). Such diffusion ratios revealed a pattern in which the constraining effect of the peptide on the lateral diffusion increased with the proportion of cone-shaped lipids (Figure 6B). POPE/CL (4:1) was the lipid composition in which lateral lipid diffusion was affected the most, closely followed by those of POPE/POPG (2:1) and POPE/POPG/CL (65:30:5). Concomitantly, the lateral diffusion of the peptide was also slower in those compositions containing POPE when compared to the bilayers having POPC as the zwitterionic component, suggesting that increased frequency of dimers was contributing to the general slowing of the diffusion. In sharp contrast, lipid diffusion in the POPC/POPG (2:1) bilayer was the least affected by the peptide, followed by that of POPC/CL (4:1). In the two POPC/POPG (2:1) simulation replicas where hourglass-shaped structures formed, lipids were observed to have a slightly faster diffusion than in the simulation without pore structures. We found a correlation between bulk lipid packing and lipid lateral diffusion: the more the peptide packs the bulk lipids, the more it slows lateral diffusion in the bilayer. We speculate that peptide molecules have diffusion coefficients in the dimeric form lower than those in the monomeric form, which could influence the annular lipid shell, slowing the overall lipid diffusion. In agreement with this, we see that the changes in lipid diffusion in the tested compositions brought about by peptide addition correlate positively with the lateral diffusion of the peptides in those systems.

**Figure 6:**
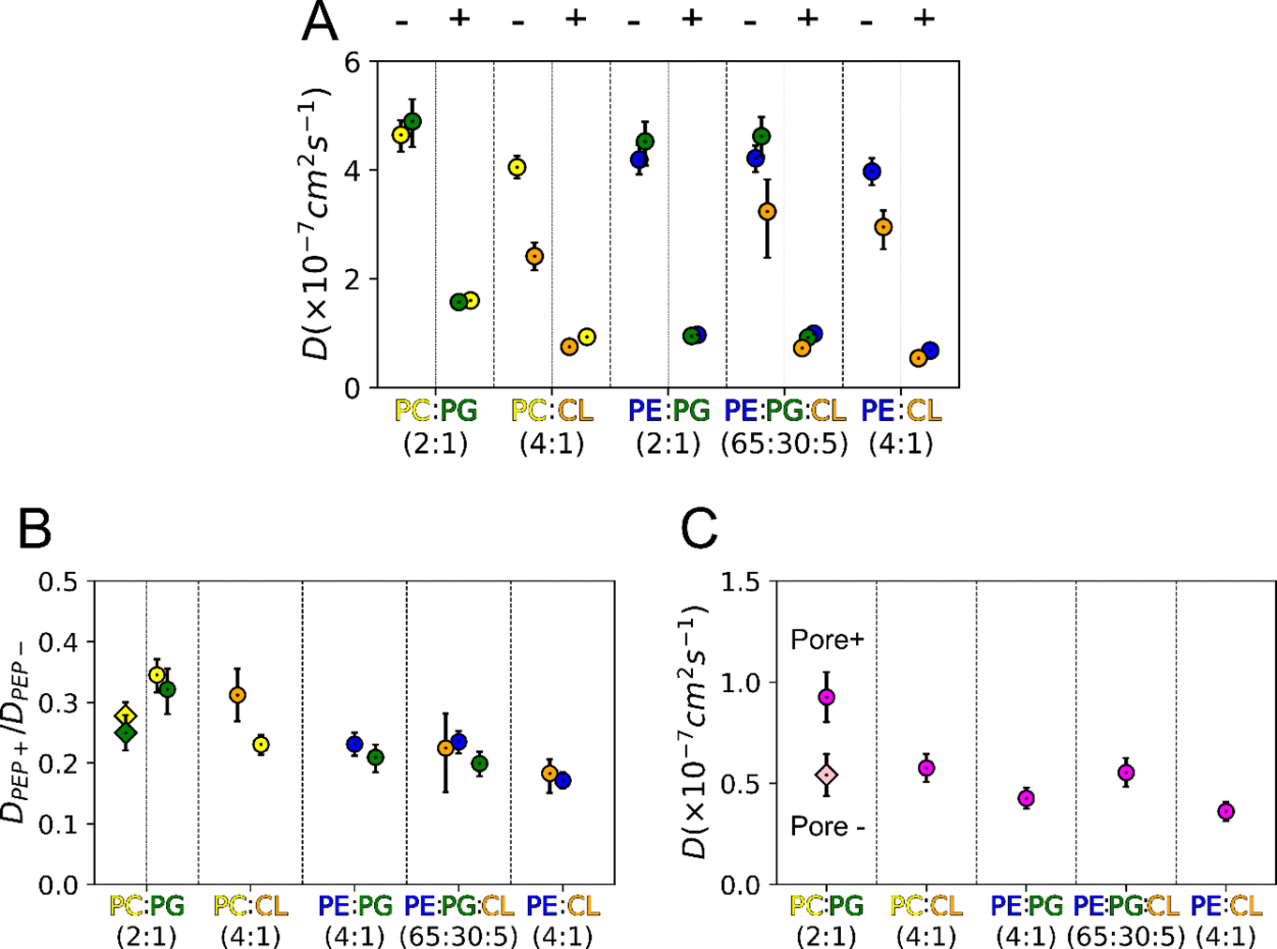
Lateral diffusion of lipids and peptides in different membrane environments. (A) Diffusion coefficients (*D*) of each lipid species in the absence (−) and presence (+) of EcDBS1R4. Error estimation is detailed in Supplementary Figure 6. (B) Ratio between the diffusion coefficients of lipids in the presence and absence of peptide (PEP+/PEP−). Diamonds in the PC/PG (2:1) bilayer represent the diffusion ratio values in the absence of pore. (C) Peptide diffusion coefficients. Lipid color codes are orange for CL, yellow for POPC, blue for POPE, and green for POPG. Error bars of parts B and C represent the propagated uncertainty of the ratios from (A).

### Peptide–Lipid Interactions

Since EcDBS1R4 has a positive net charge of +5, it was expected to engage preferentially in electrostatically driven interactions with anionic lipids. To verify whether EcDBS1R4 had preferential contacts with specific lipids, we quantified the lipid fraction (per lipid species) in contact (cutoff distance = 0.7 nm) with any peptide (here termed *f*_*c*_).

Dimerization of EcDBS1R4 results in fewer lipids available to interact with each peptide, as the contact surface per peptide molecule available for interaction with lipids decreases in dimers relative to monomers. This is visible in the plots of *f*_*c*_ over time (Supplementary Figure 7), in which *f*_*c*_ decreases quickly or gradually for all lipids. The increased dimerization propensity of EcDBS1R4 in bilayers with larger proportions of cone-shaped lipids further lowers the average *f*_*c*_.

Consistently with a preference of EcDBS1R4 for anionic lipids, for all simulated compositions, the *f*_*c*_ of zwitterionic lipids rapidly decreases in the first 3–5 μs. In contrast, for anionic lipids, this decrease is less dramatic (Supplementary Figure 7).

We calculated the amount of lipids solvating each peptide, or lipids per peptide (LpP), for each lipid species using a 0.7 nm distance threshold (Figure 7A). Considering the proportions of each lipid on a given lipid bilayer, one can estimate how many of those solvating lipids are of each species, assuming a random lipid distribution. We termed this naive estimate LpP_EXPECTED_. The ratio between the observed and expected lipids per peptide (LpP_OBSERVED_/LpP_EXPECTED_) was used as a metric of the relative affinity of each lipid species to the peptide (Figure 7B). As expected, EcDBS1R4 has higher LpP_OBSERVED_/LpP_EXPECTED_ ratios for anionic lipids than does it for the zwitterionic ones. The LpP_OBSERVED_/LpP_EXPECTED_ ratio of all zwitterionic lipids was below 1.0 (indicating fewer lipid-peptide contacts compared to a random distribution), while anionic lipids generally had LpP_OBSERVED_/LpP_EXPECTED_ values above 1. Interestingly, in the Gram-negative bacteria inner membrane-like composition (POPE/POPG/CL (65:30:5)), the affinity toward CL was higher than toward the also anionic POPG. To determine if this would also occur in a bilayer without zwitterionic lipids, we simulated an all-anionic, POPG/CL (4:1) bilayer. We found that POPG had an LpP_OBSERVED_/LpP_EXPECTED_ value slightly below 1 vs a LpP value above 1 for CL. This indicates that CL can outcompete POPG in terms of interactions with EcDBS1R4, even in the absence of zwitterionic lipids.

**Figure 7:**
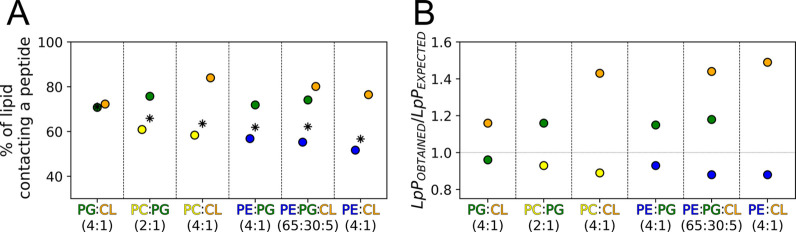
Peptide–lipid interactions in different lipid environments. (A) Percentage of lipids interacting with a peptide in each lipid bilayer. Asterisks represent the weighted mean of that percentage for all of the lipids in each bilayer. (B) Ratios of observed and expected lipid solvating a peptide (LpP). Values above 1 represent enriched interactions and vice versa. Lipid color codes are orange for CL, yellow for POPC, blue for POPE, and green for POPG.

Again, we were curious whether dimers would promote a lipid annulus composition different from that of monomers. To investigate this, we determined the ratios of lipid species contacting dimers and monomers (Supplementary Figure 8). As expected, the LpP of dimers was around half that of the monomer, since peptides in a dimer end up sharing most of their contacting lipids. Interestingly, the LpP of CL is always the most enriched in the systems where it is present, and in PC/CL and PE/PG/CL even exceeded 0.5, indicating its further enrichment in the environment of the dimer vs the monomer.

We performed a clustering-based analysis on the normalized map of contacts (cutoff distance = 0.7 nm) between each residue and the phosphates of each lipid species for the different membrane compositions (Supplementary Figure 9). From the dendrogram, it can be inferred that POPE/CL (4:1) has a unique pattern of interactions between lipids and peptide residues.

### Peptide-Induced Lipid Clustering

As previously mentioned, the proportion of zwitterionic lipids contacting a peptide decreased during the first 2–5 μs (Supplementary Figure 7). This suggests that as the peptide is surrounded preferentially by anionic phospholipids, zwitterionic lipids become progressively excluded from interacting with the peptide. We investigated whether this could result in the demixing of zwitterionic phospholipids. Radial distribution of the phosphates of the main lipid species of each composition as a function of the distance to that same lipid type (cis-RDF) strongly suggested that this was the case, as peptide addition caused an increase in the proportion of zwitterionic lipids as first and second neighbors (Figure 8A). We performed community structure measurements using interlipid distances as graph nodes^33^ to further investigate this. Distance cutoffs were defined as 2.25 times the occupancy radius of each lipid species obtained from the ApL (e.g., ∼ 1 nm for POPE in the absence of peptide). First, we measured the distribution of the cluster sizes in peptide-free bilayers (Figure 8B), showing that the POPE/POPG (2:1) mixture formed the largest clusters (mainly composed of POPE) in the absence of peptide (e.g., POPE spent 32% of the trajectory forming clusters agglutinating 10–15% of the total POPE). In contrast, compositions with CL tended to have smaller clusters in the absence of a peptide. In the purely anionic POPG/CL (4:1), POPG formed the smallest clusters (9.3% of the trajectory, forming clusters agglutinating 10–15% of the POPG).

The addition of peptide caused further lateral segregation of lipids and a concomitant increase in lipid cluster size. This clustering occurred in a composition-dependent manner, with PE-containing mixtures segregating the most (Figure 8B; the cluster that increased the most in size was that of POPE in POPE/CL (4:1) bilayers).

**Figure 8:**
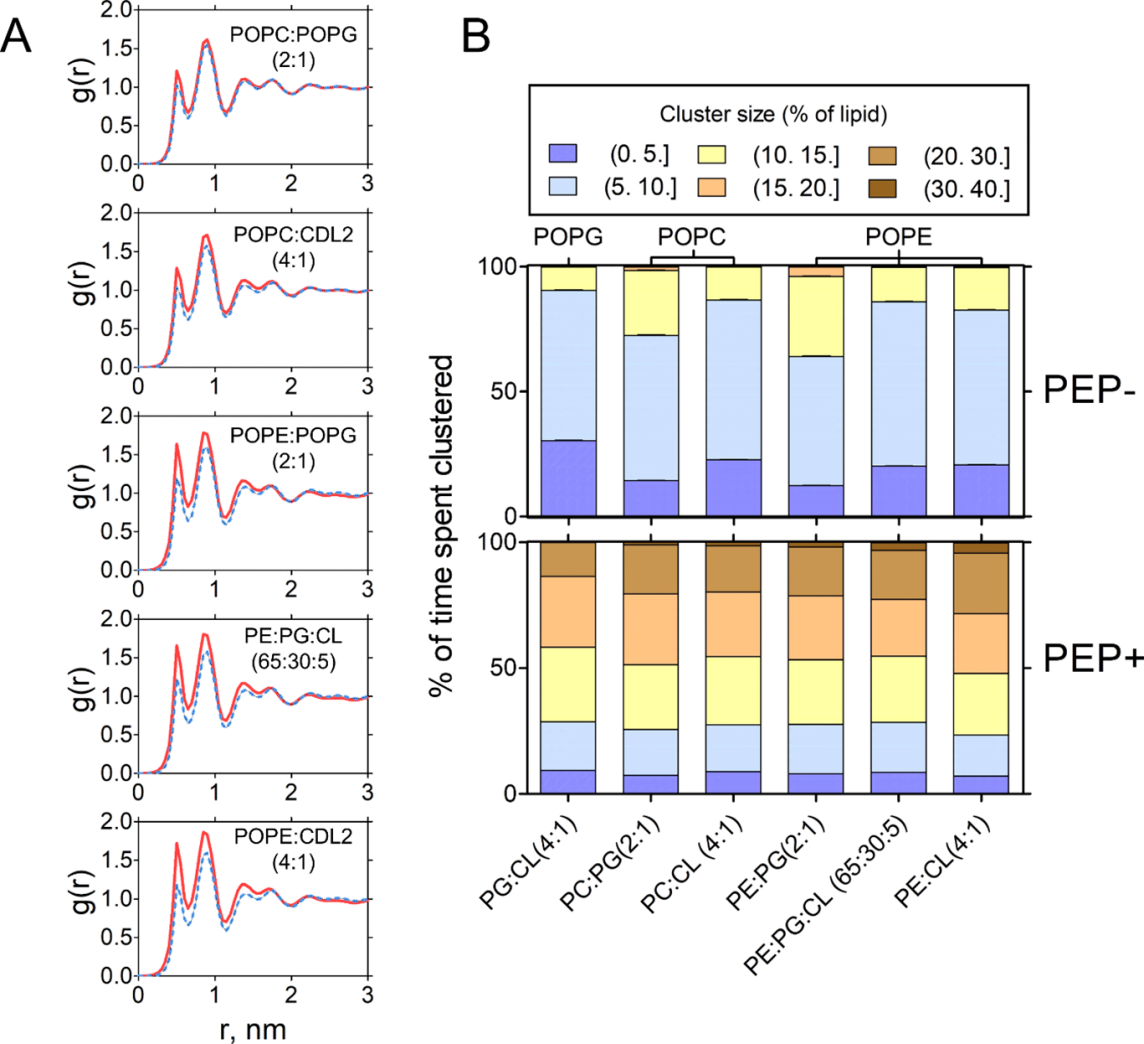
Lateral segregation of zwitterionic lipids by EcDBS1R4. (A) Radial distribution of the phosphate beads of zwitterionic lipids (either POPC or POPE) in each lipid composition as a function of the distance (*r*) to other zwitterionic lipids in the absence (blue) and presence (red) of the peptide. (B) Distribution of cluster sizes of the major lipid species in each composition in the absence (top) and presence (bottom) of peptides.

## Discussion

We employed coarse-grained molecular dynamics simulations to systematically study the effect of the lipid shape on the mode of action of a model cationic amphipathic AMP, EcDBS1R4. Our results provide mechanistic insights on how the membrane activity of a model AMP is modulated by different lipid environments. In the packed lipid environment of cone-shaped lipid-rich bilayers, EcDBS1R4 tends to be less buried along the bilayer normal. We argue that such bilayers could be protected from being “pierced” by certain AMPs. An additional consequence of the shallower positioning of the peptides is an increased tendency toward dimerization. This is, to the best of our knowledge, the first report of such sharp differences in dimerization due to the lipid environment.

### EcDBS1R4 Forms Hourglass-Shaped Pores and Antiparallel Dimers

We found that under certain conditions dictated by the lipid environment, EcDBS1R4 formed hourglass-shaped pore structures that were able to conduct water (Figure 2A and Supplementary Movie 1). Despite some similarities in peptide configuration, these pores could not be classified as toroidal nor disordered toroidal, as (i) the headgroup of lipids did not line the pore together with the peptides, and (ii) lipids did not establish a toroidal topology connecting the two leaflets (Figure 2A). Conversely, the pores also did not conform to the barrel-stave peptide organization model, given the peptides’ disordered participation in the pore assembly, where each peptide is neither fully membrane-spanning nor parallel to the other peptides or the bilayer normal (Supplementary Figure 2, inset). We hypothesize that this hourglass-shaped organization may result from the very high peptide density at the pore, which could preclude the presence of lipid headgroups in the pore lumen.

The more prevalent type of self-assembly of EcDBS1R4 was antiparallel dimers. Dimerization of EcDBS1R4 is driven by electrostatic interactions (Lys4–C-terminus) and by a segregation effect, whereby the many Lys/Arg residues spread out (presumably due to charge repulsion and to contact anionic lipids), which then exposes the EcDBS1R4 apolar face for self-interaction.

### Pore and Dimer Formation Propensities Are Dictated by Lipid Shape

Our research strategy was built upon a large body of experimental and theoretical works from the early research in AMP–lipid interactions.^12,34,35^ The use of molecular shape as a conceptual framework to address the interactions between AMPs and lipids in a lipid bilayer has proven a fruitful strategy to predict the membrane activity of AMPs.^12,34^ According to the molecular shape perspective, when an amphipathic peptide interfacially interacts with a lipid bilayer, it acts as a wedge, displacing the headgroups apart and generating a “void” at the level of the acyl chains. This void must be filled with the tail acyl chains of neighboring lipids, and this process may result in the generation of local curvature deformations, a pivotal point that can determine the membrane activity of the peptide. The cone-shaped zwitterionic phosphatidylethanolamine (PE) reduces the packing frustration exerted by the interfacially interacting amphipathic peptide by adjusting the acyl chain bond orientation to a greater extent than its zwitterionic, cylinder-shaped counterpart phosphatidylcholine (PC) (Supplementary Figure 2B). We argue that this feature provides bilayers rich in POPE with a packing buffer, which maintains the peptide in a shallower position, hindering transmembrane interactions and pore formation.^36^

Extensive research has examined the effect that peptide dimerization has on AMP activity. In some AMPs, dimerization leads to greater activity, frequently associated with enhanced membrane permeabilization.^37^ Cross-linked magainin 2 dimers, for instance, induce larger and longer-lasting pores than monomers, albeit at the expense of a lower rate of pore formation,^38^ since oligomers of higher order seem to enhance optimal pore formation.^39^ In contrast, other studies have concluded that AMP dimerization may cause unwanted hemolysis^40^ or decrease growth inhibitory activity.^41^ We report that the dimerization propensity of EcDBS1R4 increases in the presence of cone-shaped lipids. Such negative-curvature lipids, having a lower lateral pressure in the interfacial region, presumably facilitate the adsorption of the bulkier peptide dimers. Likely owing to this, dimers consistently became enriched in CL neighbors vs monomers. However, factors such as the combination with anionic character might also be at play, since no enrichment in the also-conic POPE was observed (Supplementary Figure 8). These results are in good agreement with, and further explain, previous experimental observations, where at high peptide-to-lipid ratios (P/L), the fluorescence of the intrinsically fluorescent C-terminal Trp19 residue of EcDBS1R4 was seen to depend on the lipid shape of its environment:^25^ it increases in the presence of POPC/POPG (7:3) vesicles but decreases in POPE/POPG/CL (65:30:5) vesicles due to a self-quenching effect indicative of self-association. From our observations in this work, such a fluorescence behavior can now be explained by the distinct dimerization propensity of EcDBS1R4 in different lipid environments. From a photophysical perspective, the observed self-quenching also has a likely explanation: in our dimer contact maps (Figure 2A,B), the most frequent interpeptide contact in mixtures containing POPE occurs between Trp19 and Lys4. Lysine residues are effective quenchers of the intrinsic fluorescence of tryptophan residues^42^ and are further present in EcDBS1R4 close to this contact at positions 3 and 5. In compositions lacking POPE, dimerization and the Trp19–Lys4 contact occurred less frequently and were, on average, shorter-lived.

We saw that the membrane charge (i.e., the proportion of anionic and zwitterionic lipids) can modulate the propensity to dimerize the dimer structure. In the purely anionic mixture of POPG/CL (4:1), EcDBS1R4 had the lowest dimer lifetime among the lipid compositions tested. This mixture has occasionally been used to mimic the lipid composition of Gram-positive bacteria such as *Staphylococcus aureus*, which was found to be immune to the action of EcDBS1R4.^43^ Interestingly, in pure POPE (zwitterionic) bilayers, the dimers were frequent and long-lasting, with their dimerization shifted toward a more completely antiparallel configuration, in which Trp19 contacts preferentially Pro1. The more complete antiparallel alignment between dimeric peptides results in a larger dimerization interface (Figure 3C), which could result in dimer stabilization. By contrast, an anionic environment may promote a shifted antiparallel configuration by prompting the C-terminal anionic charge on Trp19 to become shielded by Lys4, which somewhat frustrates other dimerization interactions.

We found that peptide dimerization influenced peptide and lipid dynamics in the bilayer, significantly slowing lipid diffusion, with a concomitant increase in bulk lipid packing. Peptide-induced hindrance of lipid lateral mobility is in agreement with previous experimental measurements for this^26^ and other peptides.^44,45^ A slowing in overall lipid diffusion may induce a toxic effect on its own, considering that lipids constitute the matrix supporting membrane proteins and associated functions. Furthermore, lipids and proteins in biomembranes can laterally organize in domains,^46^ which has been speculated to have an important role in fine-tuning the tempo-spatial organization of several biological processes.^47–49^ By altering lipid dynamics and organization, AMP oligomerization will likely impair biomembrane function, even at concentrations where membrane integrity is not yet compromised.

### Lipid Demixing

The cationic EcDBS1R4 tends to become surrounded preferentially by anionic lipids, especially CL. This prediction is backed by the experimental observation of micrometer-sized CL-enriched clusters in supported lipid bilayers made from POPC/CL (4:1).^26^ This selective attraction by AMPs, coupled with their dimerization, causes zwitterionic phospholipids to be excluded from interacting with the peptides and the growth of zwitterionic lipid clusters. This can contribute not only to the modulation of lipid diffusion, as discussed, but importantly, it can diminish the availability of anionic lipids for engaging in potential functional interactions with membrane proteins.^50,51^ Indeed, the hydrolytic activity of the *E. coli* ATP synthase was turned down by EcDBS1R4.^26^

### Biological Implications

EcDBS1R4 is efficacious against *E. coli*. *E. coli* has a high prevalence in the mammalian intestine.^52^ The microbiota homeostasis of the intestine is controlled by Paneth and intestinal epithelial cells through the excretion of AMPs.^53,54^ Due to the high selective pressure exerted by this AMP-rich habitat, the physiology of *E. coli* must be well adapted to survive. This raises the question of whether the abundance of nonlamellar lipids in its inner membrane could have evolved to protect *E. coli* from the membranolytic activity of host AMPs, which is an idea that has some experimental evidence.^55,56^ This notion also might explain the apparent paradox of the abundance of nonbilayer lipids in the *E. coli* inner membrane.^16^ Other prominent bacterial groups inhabiting the intestine^57^ also have high proportions of nonlamellar lipids,^58^ including bacteria of the genera *Streptococcus*,^59^ *Haemophilus*,^60^ *Veillonella*,^61^ *Clostridium*,^62^ *Actinomyces*,^63^ or *Bifidobacterium.* ^59^

### Caveats and Limitations

We note that the initial setup of the simulations, in which peptides are adsorbed onto both leaflets, may seem unrealistic and, thus, that certain behaviors we reported—namely, the formation of hourglass-shaped pore structures—may occur less frequently in systems with one-sided peptide distributions. We opted for this strategy to prevent an imbalance of leaflet tension, and associated curvature defects that would form had an asymmetric or one-sided peptide distribution been used.^64^ It is also possible and likely that a membrane-active peptide such as EcDBS1R4 could translocate across leaflets, thereby equilibrating inner and outer leaflet populations. Regarding a potential imbalance in leaflet tension, it should also be emphasized that in the context of bacterial cells, the crowded intracellular milieu and the cytoskeleton could counterbalance the curvature defects that may arise from the asymmetric periplasmic interaction of AMPs.

Another assumption is a constant membrane-bound peptide concentration for all lipid bilayer systems. From the partition coefficient estimations previously performed,^25^ we know that some systems will accept more peptide than others. However, for the sake of comparison across compositions, we decided on this approach to avoid adding another variable to our study.

One aspect that should be mentioned is the apparent resistance of Martini 2 bilayer models to pore formation.^65,66^ Due to that, the observed propensities for transmembrane contacts and pore formation should be interpreted in a relative rather than absolute sense: even though we only observed pores in two systems, in reality, they will probably form more readily and possibly in all the systems we addressed. It is still remarkable, however, that pore structures were observed with Martini 2. That this was only observed in the absence of cone-shaped lipids strongly suggests that these lipids hinder the emergence of transmembrane behavior. In this regard, future work with the meantime released Martini 3 model may better capture the genesis and dynamics of pore formation and other peptide–peptide interactions.^66^

## Methods

### Simulated Systems

In this work, we used the Martini 2.2 force field and the previously parametrized coarse-grained models of lipids and solvent.^67,68^ This work was initially planned with Martini 2.2 models, which was the latest Martini version at the time. After Martini 3 was published, a decision was made not to switch models since it has been shown that the dimerization and transmembrane behavior of helical peptides—key aspects in our observations—may be incorrectly represented under Martini 3.^69^ To build the initial atomistic structures of the peptide, we used the Avogadro software, assuming an α-helical secondary structure.^70^ To generate the Martini topology of the peptide, we used the script *martinize.* ^71^ To neutralize charges, we added the corresponding number of counterions, together with an added ionic strength of 150 mM of both Na^+^ and Cl^–^ ions (a salt concentration equivalent to the one used in previous experiments^25,26^ ) for all simulations. Membranes were built using the *insane* script.^72^ Assembled systems were energy-minimized, equilibrated for 50 ns, and simulated for a minimum of 30 μs (but most for 60 μs; see Table 1). Peptides were equilibrated onto membranes using a combination of two restraint strategies that allow for optimal lipid interaction (restraint 1, along the *z*-axis) while preventing untimely peptide–peptide contacts (restraint 2, *xy*-pinning).^73^ A representative snapshot of the initial configuration of the peptides can be found in Figure 1. Replicates were generated at the equilibration phase, each run with different random starting particle velocities.

### Simulation Parameters

All simulations were performed using the GROMACS software version 2020.5.^74^ Temperature was coupled (coupling time 1.0 ps) to 300 K using the v-rescale thermostat. Pressure was coupled using a Parrinello–Rahman barostat (coupling time of 12 ps and compressibility of 3 × 10^–4^), using a semiisotropic coupling scheme in which the lateral and perpendicular pressures were coupled independently to 1 bar. Typical settings for Martini simulations were used:^75^ a time step of 20 fs and nonbonded interactions with a 1.1 nm cutoff (pair-list update frequency of once per 10 steps). Coulombic interactions were computed with an implicit dielectric constant of 15.

### Analysis

The first 5 μs of all production runs were ignored for analysis. Given that the systems start out from contrived configurations—with peptides separated but aligned roughly parallel to one another (Figure 1)—the time scale for peptide diffusion and equilibration as antiparallel dimers is in the microsecond scale. In agreement with this, lipid organization around the peptides also stabilizes in this time scale (see Figure S6). Analyses were performed using an in-house analysis code, developed with extensive use of NumPy^76^ and MDAnalysis^77^ Python packages. Values are reported as averages over the analyzed time (15 last μs), with 95% confidence intervals estimated using a bootstrap procedure with 1000 resamplings. The underestimation of uncertainty due to time-correlated data was corrected for by bootstrap-resampling a lower number of data points.^78^

Peptide dimerization was analyzed by counting backbone–backbone contacts at a 0.6 nm distance cutoff. Two peptides were counted as being in a dimer when at least two residues in each peptide were in contact with the other peptide. Peptide–peptide contact maps revealed that the preferred configuration of the dimer was antiparallel, and the residue pair with the most frequent contacts was Lys4–Trp19. Thus, focus was put on these two residues to analyze the dynamics of association and dissociation of the dimers. To analyze ApL, we used a Voronoi tessellation-based method. The coordinates of the first tail bead (C1) of each acyl chain were used, projected onto the *xy* plane, as the centers from which the Voronoi diagrams were generated.^79^ For ApL measurement of lipids bordering peptide pore structures, Voronoi cells were defined against selected peptide particles (the Trp19 backbone beads) also projected onto the *xy* plane. Lipid clustering was measured using a community structure-based method,^80^ using a fast-greed algorithm for community detection. An interlipid distance of 2.25 times the radius of the lipid (obtained previously in the ApL measurements in the absence of peptide) was used as a distance cutoff to form the graph nodes.

## Supplementary Material





## Data Availability

The models, structures, and simulation parameters (in GROMACS format) used in this work, as well as analysis/plotting code and the data that was presented, have been publicly deposited in the Zenodo repository with DOI 10.5281/zenodo.14201669.

## Notes

Additional analyses in control systems, discriminated by peptide state (dimer/monomer), and discriminated per replica; contact clustering analysis (PDF)
Side and top views of a water crossing event through an EcDBS1R4 hourglass-shaped pore in a POPC/POPG (2:1) lipid bilayer; movie duration corresponds to 290 ns of simulation time (ZIP)
