# Supplementary material for: Lipid Shape as a Membrane Activity Modulator of a Fusogenic Antimicrobial Peptide
Source: J Chem Inf Model. 2025 Mar 20;65(9):4554–67. doi: 10.1021/acs.jcim.4c02020 (PMC12818768; doi:10.1021/acs.jcim.4c02020)
Supplement: Supplementary file 1 [file ci4c02020_si_001.pdf]

Supporting information for

Lipid shape as a membrane activity modulator of a fusogenic antimicrobial peptide

Marcin Makowski[1,2,3,4,5,6], Octávio L. Franco[7,8,9], Nuno C. Santos[1,2,*], Manuel N. Melo[3,*]

[1] GIMM – Gulbenkian Institute for Molecular Medicine, Av. Prof. Egas Moniz, 1649-035 Lisbon, Portugal

[2] Faculdade de Medicina, Universidade de Lisboa, Av. Prof. Egas Moniz, 1649-028 Lisbon, Portugal

[3] Instituto de Tecnologia Química e Biológica António Xavier, Universidade Nova de Lisboa, Oeiras, Portugal

[4] Facultad de Ciencias Químicas, Departamento de Química Física, Universidad Complutense de Madrid, Avda. Complutense s/n, Madrid, 28040 Spain.

[5] Instituto de Investigación Biomédica Hospital Doce de Octubre (imas12), Avenida de Córdoba s/n, Madrid, 28041 Spain

[6] Instituto Pluridisciplinar, Paseo Juan XXIII 1, Madrid, 28040 Spain

[7] Programa de Pós-Graduação em Patologia Molecular, Faculdade de Medicina, Universidade de Brasília, Campus Darcy Ribeiro, Asa Norte, Brasília, DF 70910900, Brazil

[8] Centro de Análises Proteômicas e Bioquímicas, Pós-Graduação em Ciências Genômicas e Biotecnologia, Universidade Católica de Brasília, SGAN 916 Módulo B, Asa Norte, Brasília, DF 70790160, Brazil

[9] S-inova Biotech, Programa de Pós-Graduação em Biotecnologia, Universidade Católica Dom Bosco Avenida Tamandaré 6000, Campo Grande, MS 79117900, Brazil

* Corresponding authors:

Manuel N. Melo (m.n.melo@itqb.unl.pt)

Nuno C. Santos (nsantos@medicina.ulisboa.pt; nuno.santos@gimm.pt)

Supplementary Movie 1. Side- and top-views of a water crossing event through an EcDBS1R4 hourglass-shaped pore in a POPC:POPG (2:1) lipid bilayer. Movie duration corresponds to 290 ns of simulation time.

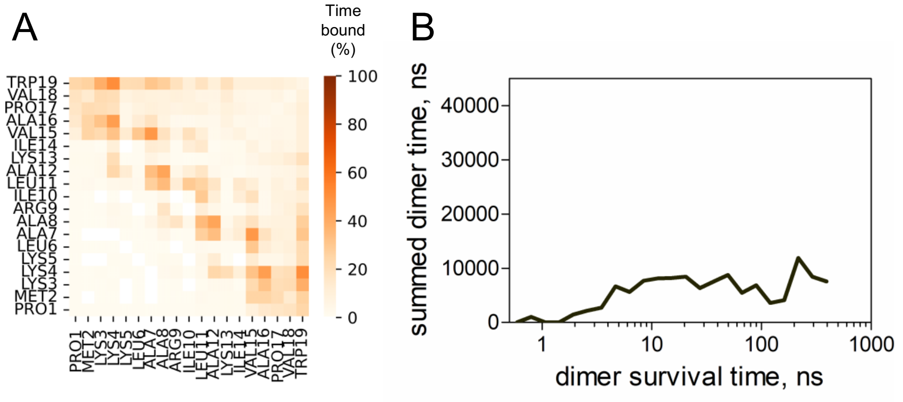

Supplementary Figure 1. EcDBS1R4 dimerization tendency in a pure POPE lipid bilayer. Note that to directly compare lifetimes with those of Figure 2B, the Y-values should be scaled by 2×, since the simulation time for this composition was 10 μs per replicate, contrasting with the 20 μs for the compositions depicted in Figure 2B.

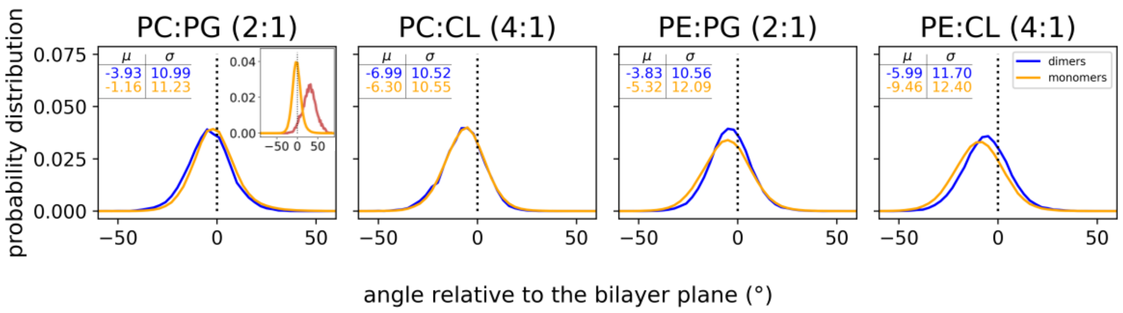

Supplementary Figure 2. Peptide insertion angle distributions of EcDBS1R4 in monomeric or dimeric assembly (orange and blue, respectively). Positive angles imply an orientation in which the C-terminal end of the peptide is buried deeper than the N-terminal, and vice-versa. PC:PG (2:1) mixture (inset): insertion angle distribution for peptides participating in pore structures (maroon) and excluded from the pore structure in monomeric form (orange).

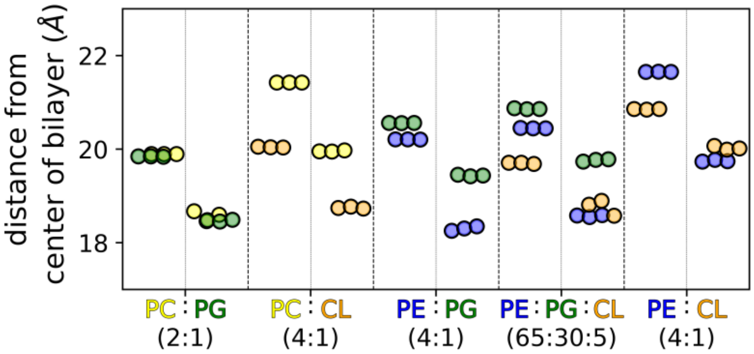

Supplementary Figure 3. Effect of EcDBS1R4 on bilayer thickness. For each replicate, average distance of the phosphate bead of each lipid species from the bilayer center in the absence (-) and presence (+) of EcDBS1R4. Lipid color codes are orange for CL, yellow for POPC, blue for POPE, and green for POPG. Errors bars (smaller than the symbol size) are reported as 95% confidence intervals determined through bootstrapping 1000 re-samplings of block averaged data (500 equally sized blocks) from the three replicas.

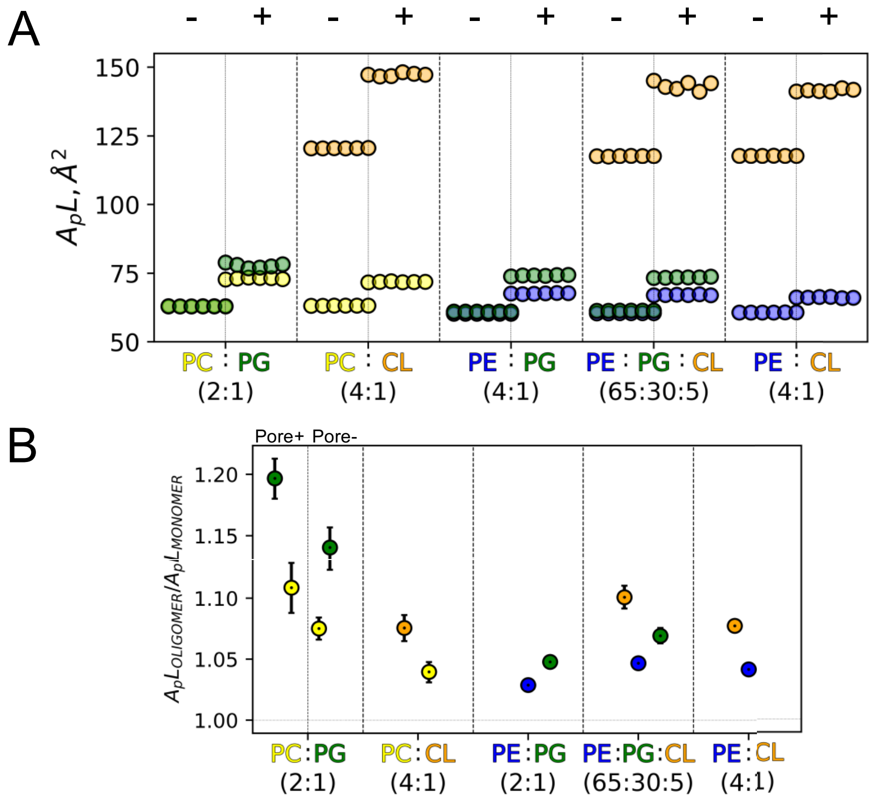

Supplementary Figure 4. (**A**) For each replicate, average area per lipid of all lipids for each lipid composition, in the absence (-) and presence (+) of EcDBS1R4 (per monolayer). Error bars (smaller than the symbol size) are reported as 95% confidence intervals determined through bootstrapping 1000 re-samplings of block-averaged ApL data (500 equally sized blocks) from the three replicas. (**B**) Ratio of the area per lipid (ApL) of annular lipids surrounding a peptide oligomer (either in dimer or pore structure) to that of a peptide monomer. For the PC:PG (2:1) composition, the ApL ratios for pore structures and monomers are shown on the left and right sides of the corresponding column, respectively. Values above 1 (indicated by the horizontal dashed line) signify an increase in the ApL of annular lipids in the dimer relative to the monomer. Lipid color codes are orange for CL, yellow for POPC, blue for POPE, and green for POPG.

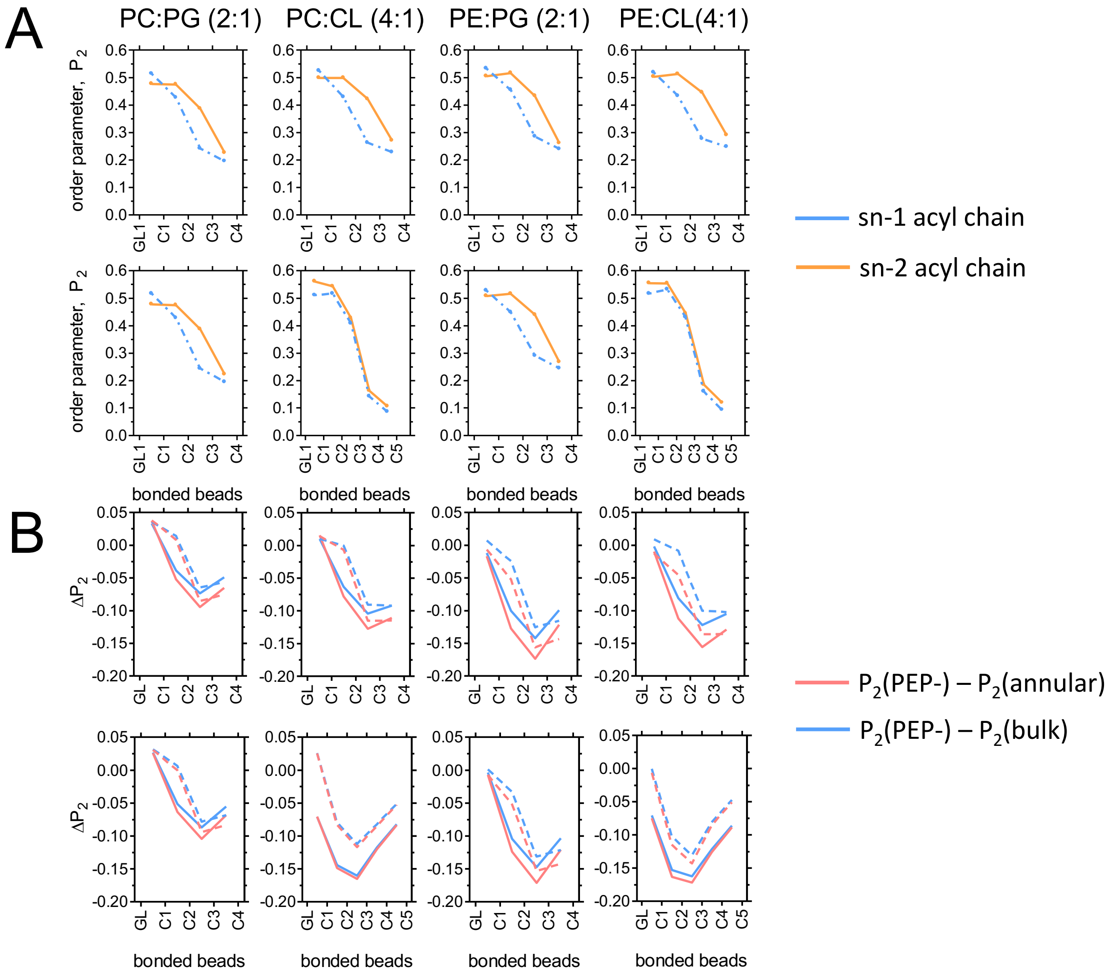

Supplementary Figure 5. Effects of EcDBS1R4 on acyl chain order. (**A**) P_2 bond-orientational order parameter of the bonds of the sn1 (blue) and sn2 (orange) acyl chains of the zwitterionic (top) and anionic (bottom) lipids in the control situation (no peptide). (**B**) Variation of the P_2 order parameter due to the addition of EcDBS1R4 ($\Delta P_2 = P_2^{PEP+} - P_2^{PEP-}$) in the sn1 (straight line) and sn2 (dashed) acyl chains of the bulk (blue) and annular (red), zwitterionic (top) and anionic (bottom) lipids in each composition. Negative ΔP_2 values mean that the order parameter at that bond is lower for the bilayer in the presence of the peptide than in its absence.

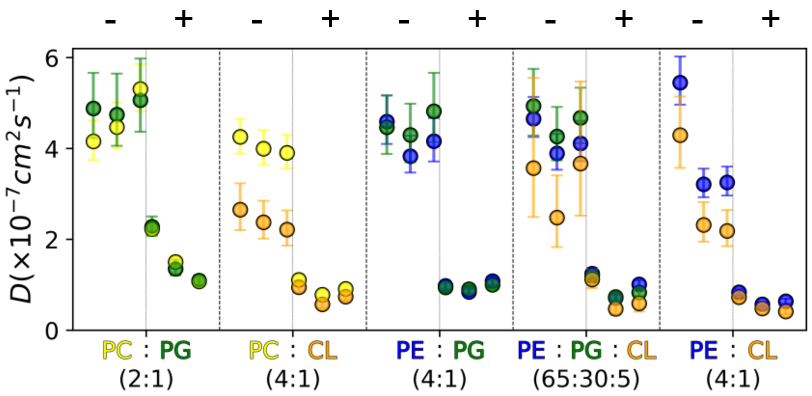

Supplementary Figure 6. Diffusion coefficients (*D*) per replicate of each lipid species in the absence (-) and presence (+) of EcDBS1R4. Lipid color codes are orange for CL, yellow for POPC, blue for POPE, and green for POPG. Errors are reported as 95% confidence intervals determined through bootstrapping (10000 re-samplings).

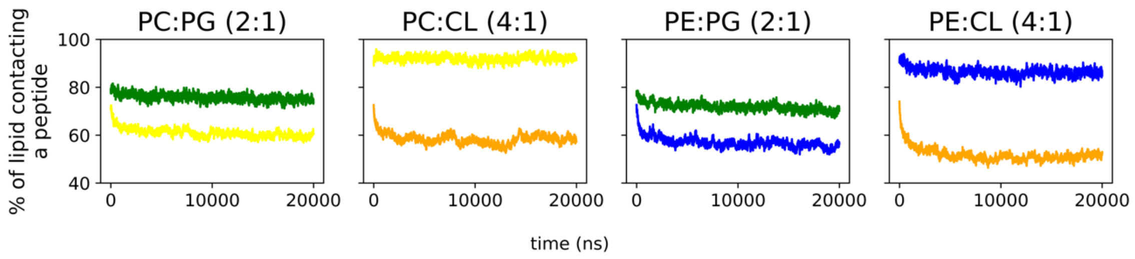

Supplementary Figure 7. Time-evolution of the percentage of each lipid species molecules in contact with any peptide, per lipid composition. Lipid color codes are orange for CL, yellow for POPC, blue for POPE, and green for POPG.

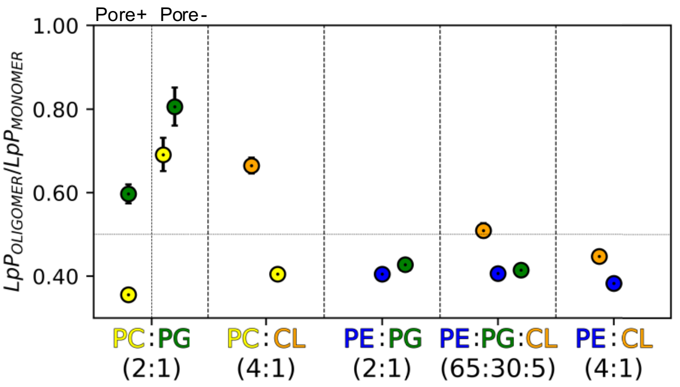

Supplementary Figure 8. Ratio of annular lipids per peptide (LpP) in an oligomeric state (either dimer or pore structure) to that in the monomeric state. For the PC:PG (2:1) composition, the LpP ratios for pore structures and monomers are shown on the left and right sides of the column, respectively. Because of the shared annular region, dimers have roughly half the lipids per peptide to interact with; as such, values above 0.5 represent an enrichment of a given lipid around a dimer relative to the monomer. This approximation breaks down for PC:PG (2:1), where dimers are less well-defined and ratios between 0.5 and 1.0 are obtained for the dimers that do form. Lipid color codes are orange for CL, yellow for POPC, blue for POPE, and green for POPG.

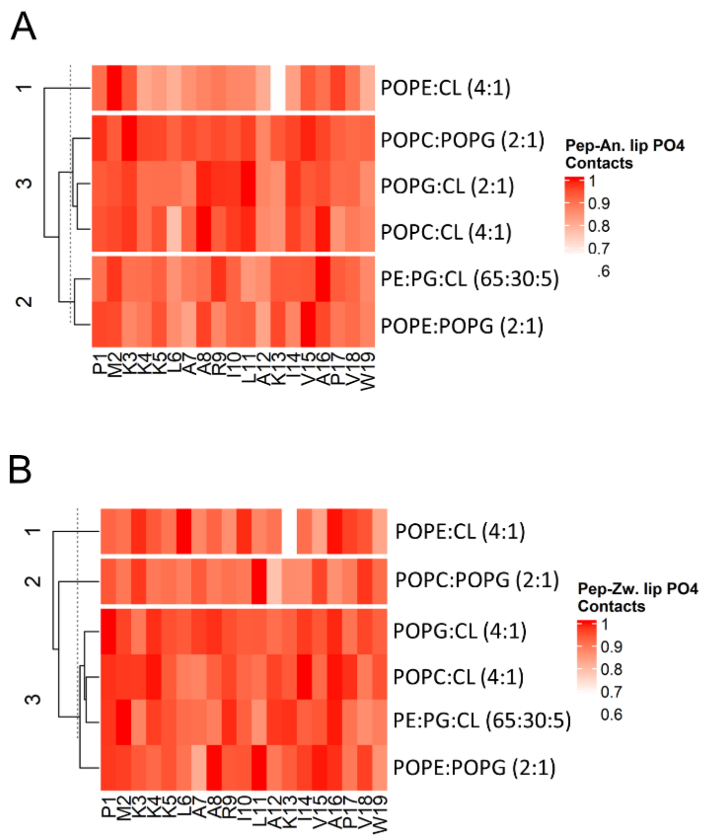

Supplementary Figure 9. Hierarchical clustering dendrograms of the interactions between peptide residues and phosphate beads of anionic (**A**) and zwitterionic (**B**) lipids in each lipid mixture.
